# Tanreqing injection inhibits dengue virus encephalitis by suppressing the activation of NLRP3 inflammasome

**DOI:** 10.1186/s13020-024-00893-2

**Published:** 2024-02-14

**Authors:** Hefei Huang, Xuemei He, Lingzhu Shi, Jingtao Yu, Zibin Lu, Huihui Cao, Jinying Ou, Xi Chen, Lijun Yan, Jiabin Yang, Wei Zhao, Junshan Liu, Linzhong Yu

**Affiliations:** 1 Third Level Research Laboratory of State Administration of Traditional Chinese Medicine, School of Traditional Chinese Medicine, Southern Medical University, Guangzhou, 510515 People’s Republic of China; 2 Guangdong Provincial Key Laboratory of Tropical Disease Research, School of Public Health, Southern Medical University, Guangzhou, 510515 People’s Republic of China; 3 Guangdong Provincial Key Laboratory of Chinese Medicine Pharmaceutics, Southern Medical University, Guangzhou, 510515 People’s Republic of China; 4 Department of Pharmacy, Zhujiang Hospital, Southern Medical University, Guangzhou, 510515 People’s Republic of China

**Keywords:** Tanreqing injection, Dengue, Encephalitis, NLRP3

## Abstract

**Background:**

Encephalitis caused by dengue virus (DENV) is considered a manifestation of severe dengue. Tanreqing injection (TRQ) is a well-known Chinese patented medicine, which has been used to treat brain-related disorders by inhibiting inflammation. Nevertheless, the effects of TRQ on DENV encephalitis have not been studied. The aim of this study was to evaluate the effects of TRQ on DENV encephalitis and to explore its potential mechanisms.

**Methods:**

The cytotoxicity of TRQ was examined by MTT assay, and the anti-DENV activities of TRQ in BHK-21 baby hamster kidney fibroblast were evaluated through CCK-8 and plaque assays. The expression levels of NO, IL1B/IL-1β, TNFα and IL6 were measured by qRT‒PCR and ELISA in the BV2 murine microglial cell line. The inhibitory effects of TRQ on NLRP3 inflammasome activation in BV2 cells were examined by Western blotting, qRT‒PCR and ELISA. The effects of TRQ on HT22 mouse hippocampal neuronal cells were examined by CCK-8 assay, morphology observation and flow cytometry. Moreover, a DENV-infected ICR suckling mouse model was developed to investigate the protective role of TRQ in vivo.

**Results:**

TRQ decreased the release of NO, IL6, TNFα and IL1B from BV2 cells and inhibited the activation of NLRP3. The presence of the NLRP3 agonist nigericin reversed the anti-inflammatory activities of TRQ. Furthermore, TRQ inhibited the death of HT22 cells by decreasing IL1B in DENV-infected BV2 cells. In addition, TRQ significantly attenuated weight loss, reduced clinical scores and extended the survival in DENV-infected ICR suckling mice. Critically, TRQ ameliorated pathological changes in ICR suckling mice brain by inhibiting microglia and NLRP3 activation and decreasing the production of inflammatory factors and the number of dead neurons.

**Conclusion:**

TRQ exerts potent inhibitory effects on dengue encephalitis in vitro and in vivo by reducing DENV-2-induced microglial activation and subsequently decreasing the inflammatory response, thereby protecting neurons. These findings demonstrate the potential of TRQ in the treatment of dengue encephalitis.

**Supplementary Information:**

The online version contains supplementary material available at 10.1186/s13020-024-00893-2.

## Introduction

Dengue is a mosquito-borne viral disease attributed to dengue virus (DENV) infection. More than half of the world’s population is at risk of dengue, with approximately 100 million cases of symptomatic dengue coming into being annually [1]. Central nervous system (CNS) disease is included in the definition of severe disease according to the World Health Organization’s ‘dengue guidelines’, such as dengue encephalitis [2]. The clinical spectrum of dengue encephalitis has expanded, and 61% of patients with neurological features of dengue belong to patients with encephalitis [3]. Patients with dengue encephalitis have a higher chance of developing dengue shock syndrome (DSS) and dengue hemorrhagic fever (DHF). DSS, dengue encephalitis and dengue encephalopathy are the most common triggers of increased morbidity and mortality [4, 5]. Currently, no dengue vaccine can produce type-specific or cross-specific neutralizing antibodies against all DENV serotypes regardless of individual immune status and age at vaccination. [6, 7]. Additionally, there are no effective antiviral drugs for symptomatic DENV infection, especially dengue encephalitis [8, 9].

Inflammation plays a crucial role during the pathogenesis of dengue infection and has been described in both animal models and humans in several studies [10]. DENV can facilitate the assembly of NLRP3 inflammasome during infection, which then regulates the cleavage of inactive pro-IL-1β (interleukin 1 beta, IL1B) via activating Caspase-1 (CASP1), a process that yields mature IL1B, thereby activating inflammation [11].

Tanreqing injection (TRQ), consisting of *Scutellaria baicalensis* Georgi (Lamiaceae), *Selenaretos thibetanus* Cuvier (Ursidae), *Capra hircus* Linnaeus (Bovidae), *Forsythia suspensa* (Thunb.) Vahl (Oleaceae) and *Lonicera japonica* Thunb (Caprifoliaceae), is a new type of Chinese medicine (TCM) with antipyretic, antiviral, antibacterial, and anti-convulsion effects [12]. The multicomponent mixture in TRQ was screened and identified by HPLC–DAD-TOF/MS and 12 compounds were confirmed by online ESI-TOF/MS [13, 14]. Among the chemical constituents of TRQ, chlorogenic acid, protocatechuic acid, caffeic acid, baicalin, ursodeoxycholic acid, chenodeoxycholic acid and forsythin was considered as the representative compounds due to their higher abundances. A previous study has confirmed the blood–brain barrier penetrance ability of chlorogenic acid, baicalin, ursodeoxycholic acid, and chenodeoxycholic acid [1]. These compounds have been shown to display antiviral effects against a variety of viruses, including influenza virus, herpes simplex virus and immunodeficiency viruses [15–23]. Moreover, these compounds have been demonstrated to exert anti-inflammatory effects to treat various inflammation-related diseases [24–27]. Clinically, TRQ is mainly used for the treatment of infantile acute pneumonia, acute upper respiratory tract infection, acute cerebral ischemia and acute cholecystitis [12, 28]. TRQ has been used in the treatment of brain-related diseases. It can also treat cerebral ischemia by regulating Ca^2+^ transport and treat migraine by regulating inflammation [29]. Therefore, we hypothesized that TRQ may alleviate dengue encephalitis. The efficacy and potential mechanisms of TRQ against DENV-induced encephalitis were explored both in vivo and in vitro in this research.

## Materials and methods

### Antibodies and reagents

TRQ is produced exclusively by Shanghai Kaibao Pharmaceutical Co., Ltd. (NO. 2003305, Shanghai, China). Antibodies against β-actin (ACTB), cleaved Caspase-1 (CASP1), pro-Caspase-1, NLRP3 and peroxidase-labeled anti-rabbit immunoglobulin were acquired from Cell Signaling Technology (Danvers, MA, USA). An Annexin V-FITC apoptosis detection kit and Hoechst 33258 were purchased from Thermo Fisher Scientific (Waltham, MA, USA). Interleukin-6 (IL6) and tumor necrosis factor-α (TNFα) enzyme-linked immunosorbent assay (ELISA) kits were acquired from Dakewei (Beijing, China). IL1B ELISA kits were purchased from Huamei (Wuhan, Hubei, China). NO kits were acquired from KeyGen BioTECH (Nanjing, Jiangshu, China). Other agents were acquired from Sigma-Aldrich (St. Louis, MO, USA). Ursodeoxycholic acid, protocatechuic acid, caffeic acid, forsythin, chenodeoxycholic acid, baicalin and chlorogenic acid were acquired from Shanghai Yuanye Bio-Technology Co., Ltd. (Shanghai, China), the lot numbers of which are M11GB140672, M27GB143417, A22GB158496, Y12S11W124066, H30N9Z76269, N15GB167969, and N05GB166572, respectively. The purities of seven standard compounds met the requirements of high-performance liquid chromatography (HPLC) analysis (≥ 98.0%). Ultrapure water was supplied by a Synergy UV-R water purification system (Millipore, Billerica, MA, USA). Formic acid was purchased from Sigma-Aldrich. HPLC grade methanol was acquired from Thermo Fisher Scientific.

### Preparation of TRQ

The quality of TRQ was evaluated by UPLC-MS–MS [13]. Chromatographic analysis was conducted on an Xevo TQ-XS Triple Quadrupole Mass Spectrometer (Waters, Milford, MA, USA) and an ACQUITY H-class plus UPLC System (Waters, Milford, MA, USA). Gradient elution was performed with 0.1% formic acid aqueous solution as solvent A and methanol as solvent B. The gradient elution was performed under the following conditions: 0–1 min, 5% B; 1–3 min, 5–95% B; 3–7 min, 95% B; 7–7.1 min, 95–5% B; 7.1–10 min, 5% B. The flow rate was set at 0.3 mL·min^−1^. The samples were then separated with an ACQUITY UPLC HSS T3 column (2.1 mm × 100 mm, 1.7 μm) (Waters, Milford, MA, USA). The temperature of the column and the automatic sampler were set at 40 ℃ and 10 ℃, respectively. The injection volume was 1 µL. Negative ion ionization was selected for the detection of all compounds.

The ESI interface was linked to triple quadrupole tandem mass spectrometric detection. The capillary voltage was − 2500 V, and the pressure of the nebulizer was 7 bar. The nebulizing and dry gas was served by high-purity nitrogen. The desolvation temperature was 550 ℃, the collision gas flow rate was 0.15 mL·min^−1^, and the desolvation gas flow rate was set at 1000 L·Hr^−1^. Multiple reaction monitoring (MRM) conditions were optimized by infusion of the reference standard, as shown in Table 1.

**Table 1:** Optimized MRM parameters for the detection of analytes

Analyte	Precursor	Product	CE/V	Polarity
Baicalin	445.0	269.0	20	Negative
Protocatechuic acid	153.0	108.9	15	Negative
Caffeic acid	178.9	134.8	15	Negative
Chlorogenic acid	353.1	191.0	15	Negative
Ursodeoxycholic acid	437.1	391.0	15	Negative
Chenodeoxycholic acid	437.1	391.0	15	Negative
Forsythin	533.2	371.2	15	Negative

Each standard substance was dissolved or diluted individually to obtain a final concentration of 1 mmol/L as a stock solution. One hundred microliters of each stock solution was transferred to a 10 mL volumetric flask to form a mixed working standard solution. All standard solutions were stored at − 20 ℃. A series of calibration standard solutions were then prepared by stepwise dilution of this mixed standard solution. The sample was diluted four times with methanol, followed by incubation at − 20 ℃ for 1 h and centrifugation at 12 000 rpm and 4 ℃ for 15 min. The supernatant was diluted 1 time, 10 times, 100 times, or 1000 times for UPLC-MS/MS analysis.

### Cell culture and virus

The mosquito larva C6/36 cells (American Type Culture Collection, ATCC, Rockville, MD, USA) were cultured in RPMI-1640 supplemented with 1% (*v*/*v*) penicillin/streptomycin (Gibco, the Netherlands) and 10% (*v*/*v*) fetal bovine serum (FBS, ExCell Bio, Shanghai, China) at 28 ℃. The baby hamster kidney fibroblast BHK-21 cells (ATCC) were cultured in RPMI-1640 supplemented with 10% (*v*/*v*) FBS [30]. The mouse hippocampal neuronal cell line HT22 was generously provided by Professor Weidong Cheng (Southern Medical University). HT22 cells and the murine microglial cell line BV2 (BeNa Culture Collection, Beijing, China) were cultured in DMEM supplemented with 10% (*v*/*v*) FBS [31, 32]. The DENV-2 New Guinea C strain was kept in our laboratory [33] and propagated on C6/36 cells. DENV-2 was kept at − 80 ℃ until use.

### Cell viability assay

A 3-[4, 5-dimethylthiazol-2-yl]-2, 5-diphenyltetrazolium bromide (MTT, Sigma, St. Louis, MO, USA) assay was used to determine cell viability [30]. BV2 cells (1 × 10^4^ cells *per* well) or BHK-21 cells (1 × 10^4^ cells *per* well) were seeded into 96-well plates and incubated at 37 ℃ for 24 h. Then, BV2 and BHK-21 cells were cultivated with various concentrations of TRQ for 24 h or 96 h and incubated by adding 10 μL of MTT solution (5 mg/mL) *per* well for another 4 h at 37 ℃. After removal of the supernatant, 100 μL of DMSO was added to each well. Finally, a microplate reader was used to measure the absorbance at 490 nm (Thermo Fisher Scientific).

### CCK-8 assay

BHK-21 cells were cultured for 24 h in 96-well plates. For virus binding and entry assay, BHK-21 cells were infected with DENV-2 and incubated with different concentrations of TRQ (1/800, 1/400, 1/200) at the same time. For viral intracellular replication assay, TRQ (1/800, 1/400, 1/200) was added after DENV-2 was completely absorbed into BHK-21 cells. After 4 days of incubation, the cell viability was assessed according to the method described for the CCK-8 solution (APE x BIO Technology LLC, Houston, TX, USA) [34]. After incubation in the dark at 37 ℃ for 2 h, the viable cells were detected by using absorbance at a 450 nm wavelength.

### Plaque assay

First, BHK-21 cells (1 × 10^5^ cells *per* well) were seeded in a 6-well plate and incubated for 24 h. Second, cells were infected with DENV-2 (10^2^ TCID_50_/mL) with or without TRQ for 1 h at 37 ℃. Two days later, new BHK-21 cells were infected with the supernatants containing progeny virus for 1 h at 37 ℃ followed by overlaying with RPMI-1640 media containing 1.2% methyl cellulose and 2% FBS. The cells were fixed with 4% formaldehyde for 15 min followed by careful removal of the solution. Finally, the plaques were developed by adding 2% crystal violet solution for 15 min [30].

### DENV infection of BV2 cells

BV2 cells were seeded in a 6-well plate at a concentration of 2 × 10^5^ cells *per* well and incubated overnight at 37 ℃. Next, cells were infected with DENV-2 (MOI = 0.5) in the presence or absence of TRQ for 1 h at 37 ℃ [35]. After infection, BV2 cells were cultured in fresh DMEM with 2% FBS for 24 h. Finally, the cells or supernatants were used for subsequent experiments.

### RNA extraction and quantitative real-time PCR (qRT‒PCR)

The sequences of the sense and antisense primers used in this study are shown in Table 2. Total RNA was extracted from RNAiso Plus (Takara, Shiga, Japan) according to the manufacturer’s instructions. The PrimeScript^™^ RT Reagent Kit with gDNA Eraser was used immediately to synthesize cDNA. The reverse transcription parameters were set according to the previous conditions in our laboratory [36]. Subsequently, qRT‒PCR analysis was accomplished on a LightCycler 96^®^ real-time PCR (Roche, Switzerland) with TB Green^™^ Premix Ex Taq ^M^ II. No-template controls were included on each plate. The mRNA expression level of each target gene was analyzed by the 2^−ΔΔCt^ method and normalized to the expression level of *β-actin (Actb)*.

**Table 2:** Primers used in the manuscript

Primer	Forward (5ʹ-3ʹ)	Reverse (5ʹ-3ʹ)
*Actb(β-actin)*	GGCTGTATTCCCCTCCATCG	CCAGTTGGTAACAATGCCATGT
*Il6*	TAGTCCTTCCTACCCCAATTTCC	TTGGTCCTTAGCCACTCCTTC
*Tnfα*	CAGGCGGTGCCTATGTCTC	CGATCACCCCGAAGTTCAGTAG
*Il1b (IL-1β)*	GAAATGCCACCTTTTGACAGTG	TGGATGCTCTCATCAGGACAG
*Nlrp3*	ATTACCCGCCCGAGAAAGG	TCGCAGCAAAGATCCACACAG

### ELISA

The protein levels of IL1B, IL6 and TNFα in cell supernatants and tissues were determined by ELISA kits based on the manufacturer’s instructions [37].

### Western blotting

BV2 cells were homogenized in lysis buffer (150 mM sodium chloride, 50 mM Tris, pH 7.5, 1 mM phosphatase inhibitor, 1 mM dithiothreitol, 1 mM sodium orthovanadate, 1 mM EDTA, 1 mM PMSF and 1% Triton X-100). Then, samples were centrifuged at 15,000 × *g* for 20 min and the supernatants were harvested [37]. A BCA kit was used to quantify the protein concentration of the supernatants. Subsequently, the proteins were separated by 10% SDS/PAGE and transferred to polyvinylidene fluoride (PVDF) membranes (Bio-Rad, Hercules, USA). After blocking with 5% (*w/v*) skim milk for 1 h at room temperature (RT), immunoblotting was performed using appropriate primary antibodies against NLRP3 (1:1000), caspase-1 (1:1000), cleaved caspase-1 (1:1000), and β-actin (1:500). Next, the membranes were incubated at 4 ℃ overnight, washed with TBS-T and then incubated with appropriate horseradish peroxidase-conjugated secondary antibodies (1:1000) for 1 h at RT. Membranes were washed with TBS-T and then detected using enhanced chemiluminescence reagent (ECL) and visualized by a FluorChem E^™^ system (ProteinSimple, San Francisco, CA, USA).

### Apoptosis assay

Apoptosis was evaluated using the annexin V-FITC apoptosis detection kit. HT22 cells were seeded in 6-well plates (2 × 10^5^ cells *per* well) for 24 h and treated with supernatant of infected BV2 cells for 48 h. Then, cells were collected and washed with phosphate-buffered saline (PBS) twice before incubating with annexin V and PI dyes at RT for 15 min. Finally, stained cells were analyzed by flow cytometry (CytoFLEX, Beckman Coulter, Fullerton, CA, USA) [38].

### Hoechst 33,258 staining

Apoptosis was observed using Hoechst 33258 staining. The prepared paraffin sections were deparaffinized, hydrated and then washed with PBS twice. Finally, slices were stained with Hoechst 33258 (10 µg/mL in PBS) at 37 ℃ for 15 min in the dark. Then the slices were sealed, and observed under an IX 53 light microscope (Olympus, Tokyo, Japan) [38].

### Animals

Seven-day-old ICR suckling mice were purchased from SPF Biotechnology Co., Ltd. (Beijing, China) and kept in a biosafety level-2 facility of the Animal Experimental Center of Guangzhou University of Chinese Medicine. The 3 R’s of ethical principles of animal experimentation was respected and fully considered during the experiments. All animal studies were carried out in compliance with the rules of the Ethics Committee of Guangzhou University of Chinese Medicine (Permit number: 20211026004).

The mice were randomly divided into 6 groups: 1. Control group; 2. DENV-2 group; 3. DENV-2 + TRQ-L (low concentration, 1.25 mL/kg); 4. DENV-2 + TRQ-M (medium concentration, 2.5 mL/kg); 5. DENV-2 + TRQ-H (high concentration, 5 mL/kg); 6. DENV-2 + Dexamethasone (Dex, 4 mL/kg). Mice were inoculated with DENV-2 intracerebrally (4 × 10^5^ plaque-forming units, PFU) [39]. Furthermore, TRQ at different concentrations or an equal volume of saline solution was administered intraperitoneally from 0 days post infection (D.P.I). The clinical scores and body weights were recorded every day. The clinical score was divided into six grades: 0 for health, 1 for minor manifestations (reduced mobility and hunched posture), 2 for limbic seizure, 3 for dyspraxia (weakness in the front or hind limbs), 4 for paralysis and 5 for death. Finally, the mice were sacrificed to collect tissues for Western blotting, qRT‒PCR, histological and immunohistochemical analysis at 6 D.P.I. The survival rates of the mice were evaluated every day until 9 D.P.I.

### Histopathological observation

The brain tissues were rinsed with cold PBS, and fixed in 4% (*v*/*v*) paraformaldehyde immediately. Twenty-four hours later, the brain tissues were dehydrated with graded ethanol and embedded in paraffin. Brain specimens were sliced into 4 μm sections. After deparaffinization and staining with hematoxylin and eosin (H&E, Yuanye Biotech, Shanghai, China), pathological changes in each tissue section were observed with an IX 53 light microscope (Olympus, Tokyo, Japan) [40].

### Immunohistochemistry and immunofluorescence

The slices were deparaffinized and dehydrated with ethanol. Then, slices were placed in boiling citrate buffer (pH = 6.0) for 10 min for antigen retrieval. Next, endogenous peroxidase was eliminated by the addition of 3% hydrogen peroxide. After blocking with 5% bovine serum albumin for 15 min, the sections were incubated with appropriate antibody overnight at 4 ℃. For immunohistochemistry, the sections were incubated with a horseradish peroxidase-labeled kit (GTVision^™^ III Detection System/Mo&Rb, Dako, Denmark) according to the manufacturer’s instructions. Finally, the slices were stained with 3, 3ʹ-diaminobenzidine for 5 min [39].

For immunofluorescence, the sections were incubated with anti-NLRP3 antibody. Bound antibodies were visualized with Alexa Fluor 568-conjugated goat anti-rabbit tyramide signal amplification (Thermo Fisher Scientific). 4ʹ, 6-Diamidino-2-phenylindole was (DAPI; Invitrogen, Grand Island, USA) used to label the nuclei. All images were observed under an IX 53 light microscope [39, 41].

### Statistical analysis

Data are presented as the mean ± standard deviation (SD) of at least three independent experiments and analyzed by one-way ANOVA with Tukey’s test. The data were analyzed with GraphPad Prism software (Version 8.0; San Diego, CA, USA). *P* < 0.05 was considered as statistically significant.

## Results

### Quantification of seven compounds in TRQ

Linear regression was performed with the ratio of the peak area of the analyte as the ordinate (Y), and the mass concentration as the abscissa (X) (Table 3). The quantification of seven compounds was calculated. The contents of seven effective components in TRQ, including chlorogenic acid, protocatechuic acid, caffeic acid, baicalin, ursodeoxycholic acid, chenodeoxycholic acid and forsythin, were 61.01 µg·mL^−1^, 7.87 µg·mL^−1^, 178.09 µg·mL^−1^, 4516.85 µg·mL^−1^, 3181.81 µg·mL^−1^, 488.82 µg·mL^−1^ and 21.68 µg·mL^−1^, respectively.

**Table 3:** The typical equations of seven components in TRQ

Analyte	Tpical equations	R^2^	LOD(nmol/L)	LOQ(nmol/L)
Baicalin	Y = 1556.71X + 27,634.3	0.9973	< 19.53	< 19.53
Protocatechuic acid	Y = 295.403X + 2417.82	0.9984	< 19.53	< 19.53
Caffeic acid	Y = 664.609X + 7105.35	0.9988	< 19.53	< 19.53
Chlorogenic acid	Y = 805.314X + 4989.78	0.9991	< 19.53	< 19.53
Ursodeoxycholic acid	Y = 2436.31X + 33,135.7	0.9955	< 19.53	< 19.53
Chenodeoxycholic acid	Y = 2120.34X + 18,792.6	0.9959	< 19.53	< 19.53
Forsythin	Y = 1.62629X + 1.89141	0.9976	< 19.53	< 19.53

### TRQ has anti-DENV effects in vitro and in vivo

**Fig. 1:**
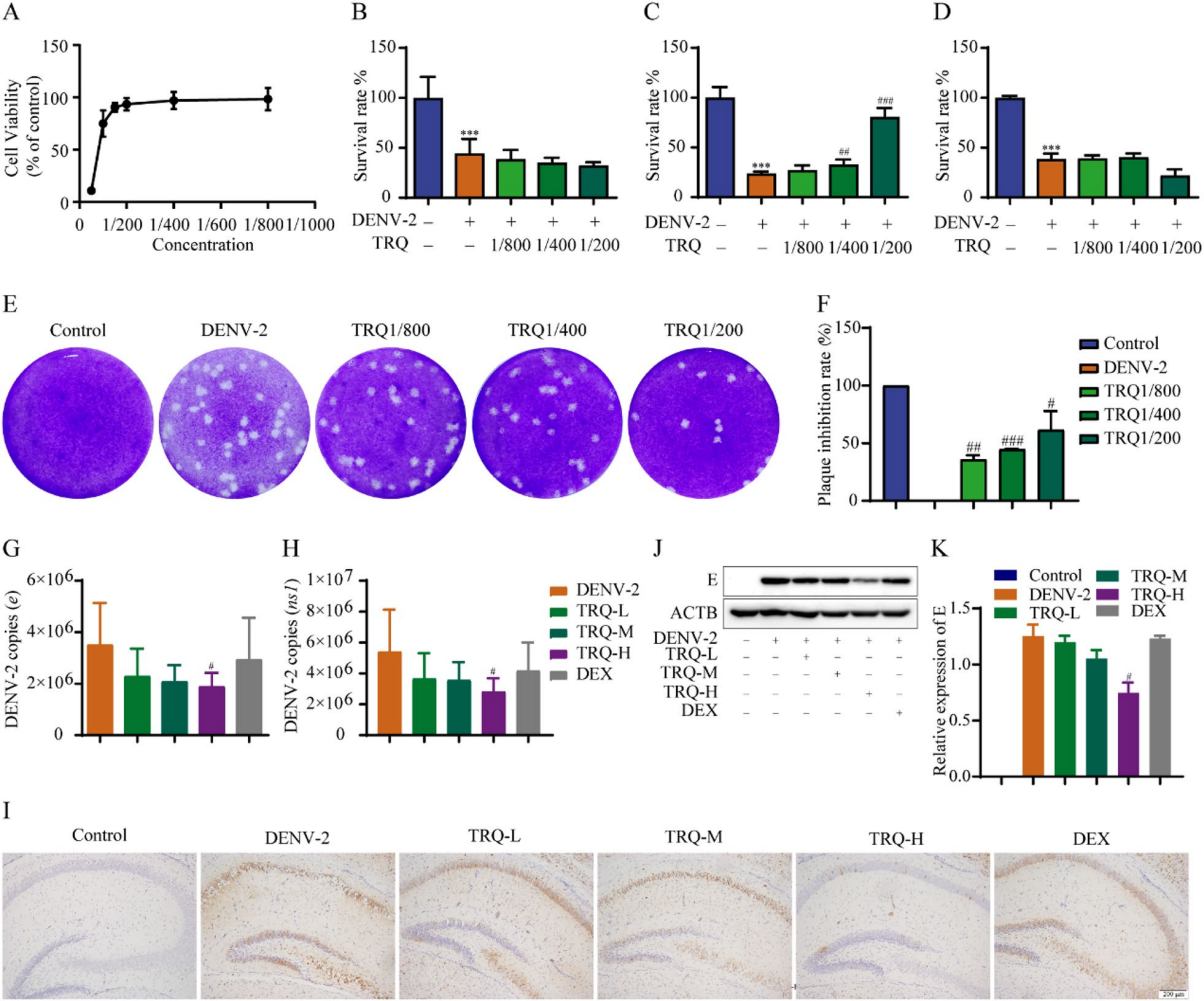
TRQ exerts anti-DENV effects in vitro and in vivo. **A** The cytotoxicity of TRQ on BHK-21 cells. BHK-21 cells were incubated with TRQ for 96 h and cell viability was determined by MTT assay. **B** TRQ shows no prophylactic anti-DENV activities, as detected by CCK-8 assay. Cells were incubated with different concentrations of TRQ and then infected with DENV-2 for 1 h. **C** TRQ exerts anti-DENV attachment/entry effects on BHK-21 cells, as detected by CCK-8 assay. BHK-21 cells were infected with 0.5 MOI of DENV-2 with or without TRQ treatment. **D** TRQ shows no anti-DENV activities after entry into BHK-21 cells, as detected by CCK-8 assay. After infection with 0.5 MOI of DENV-2 for 1 h, BHK-21 cells were treated with TRQ. **E** TRQ inhibits the production of progeny viruses in DENV-2 infected BHK-21 cells as examined by plaque assay. **F** Statistical analysis of plaque assay to verify the inhibitory effects of TRQ on DENV- induced progeny virus. **G**, **H** TRQ inhibits the viral load in the brain in DENV-2 infected ICR suckling mice at 6 D.P.I., as determined by qRT‒PCR (*n* = 6). **I–K** TRQ downregulates the protein expression level of E protein in DENV-2 infected mouse brains, as examined by immunohistochemistry (100 ×) **I**, **J** and Western blotting **K**. The concentrations of TRQ-L, TRQ-M and TRQ-H were 1.25, 2.5 and 5 mL/kg, respectively. Dex was used as a positive drug. ^***^P < 0.001 *vs*. control group. ^#^*P* < 0.05, ^##^*P* < 0.01, ^###^*P* < 0.001 *vs*. DENV-2 group

We preliminarily evaluated the inhibitory effects of TRQ on DENV. The BHK-21 cell line is extensively used to evaluate the antiviral activities of drugs. BHK-21 cells were treated with TRQ diluted to a series of concentrations. After treatment with TRQ for 96 h, no obvious toxicity was observed when the concentration was less than 1/200 (diluted 200 times) (Fig. 1A). Then, BHK-21 cells were infected with DENV-2 with or without TRQ (1/800, 1/400, 1/200), and cell viability was determined by CCK-8 assay. The results showed that TRQ at doses of 1/400, 1/200 inhibited DENV binding to cells (Fig. 1C). However, TRQ did not improve the survival rate of DENV-2 infected BHK-21 cells when administered before or after infection (Fig. 1B, D), indicating that TRQ did not show effects before and after DENV-2 entry into cells. Moreover, plaque staining results showed that TRQ significantly inhibited the plaques formed by the progeny virus (Fig. 1E–F), which indicates that TRQ decreased the production of DENV progeny virus.

We further established DENV-infected ICR suckling mice and evaluated the anti-DENV activity of TRQ in vivo. Mice were euthanized at 6 D.P.I and the brain tissues were collected. qRT‒PCR data showed that compared with the model control group, the *e* and *ns1* copies in the brain in the high-dose group was decreased after TRQ treatment, indicating that TRQ can reduce the viral load of DENV in the brain (Fig. 1G–H). Immunohistochemistry (IHC) analysis revealed that TRQ treatment, especially the high-dose group, significantly reduced DENV-induced E protein expression, which was consistent with the results of Western blotting (Fig. 1I–K). Taken together, these results demonstrate that TRQ has anti-DENV effects in vitro and in vivo.

### TRQ attenuates the release of nitric oxide (NO), IL6, and TNFα in DENV-2 infected BV2 cell*s*

**Fig. 2:**
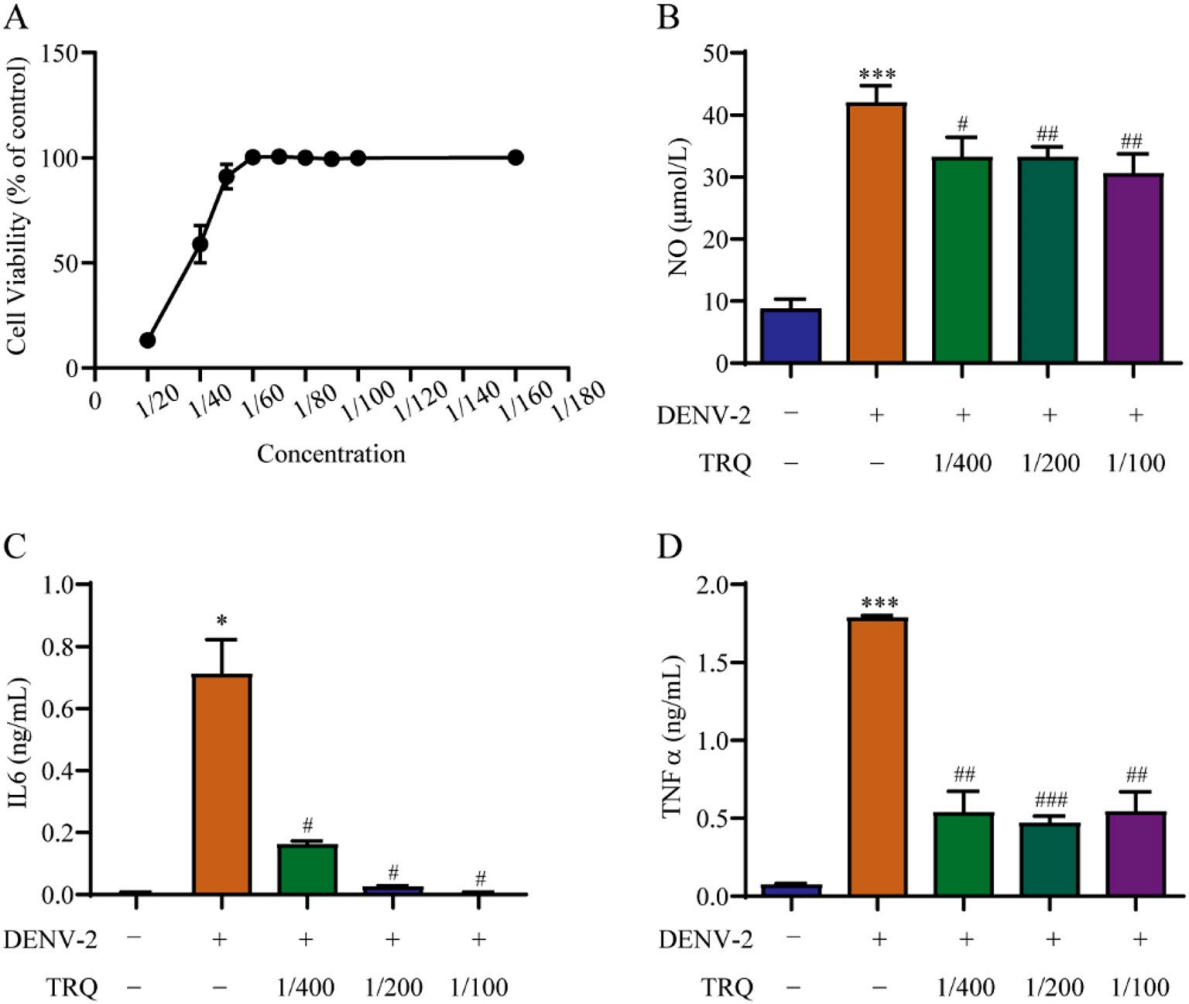
TRQ attenuates the release of NO, IL6 and TNFα in DENV-2 infected murine microglial BV2 cells. **A** The cytotoxicity of TRQ in BV2 cells. BV2 cells were incubated with TRQ for 24 h and cell viability was determined by MTT assay. **B**–**D** TRQ inhibits the release of NO, IL6 and TNFα in BV2 cells. After exposure to DENV-2 (0.5 MOI) in the presence or absence of TRQ for 24 h, the secretion of NO (**B**), IL6 (**C**) and TNFα (**D**) was measured by NO assay kits or ELISA kits. ^*^P < 0.05, ^***^P < 0.001 *vs*. control group; ^#^*P* < 0.05, ^##^*P* < 0.01, ^###^*P* < 0.001 *vs*. DENV-2 group

Encephalitis and encephalopathy are the most common neurological complications of dengue. Considering the role of inflammation in dengue encephalitis and significant anti-inflammatory effect of TRQ, we hypothesized that TRQ may alleviate DENV encephalitis by inhibiting inflammatory response. Therefore, microglial BV2 cells were selected for subsequent experiments. The qRT‒PCR data showed that the gene expression levels of *No*, *Il6* and *Tnfα* were elevated in BV2 cells after DENV-2 infection, indicating the occurrence of inflammation (Additional file 1: Fig. S1A–C). Next, we evaluated the cytotoxicity of TRQ on BV2 cells by MTT assay. As shown in Fig. 2A, no significant cytotoxicity was observed at TRQ dilution concentrations below 1/100 (diluted 100 times) for 24 h treatment. Therefore, TRQ at concentrations of 1/400, 1/200 and 1/100 was selected for the following experiments.

To explore the effects of TRQ on DENV-2-induced inflammation in BV2 cells, the release of the proinflammatory factors NO, IL6 and TNFα was detected. The results showed that TRQ inhibited the elevated levels of NO, IL6 and TNFα induced by DENV-2 in BV2 cells, which indicated that TRQ has significant anti-inflammatory effects in DENV-2 infected BV2 cells (Fig. 2B–D).

### TRQ decreases NLRP3 activation in DENV-infected BV2 cells and inhibits IL1B release

**Fig. 3:**
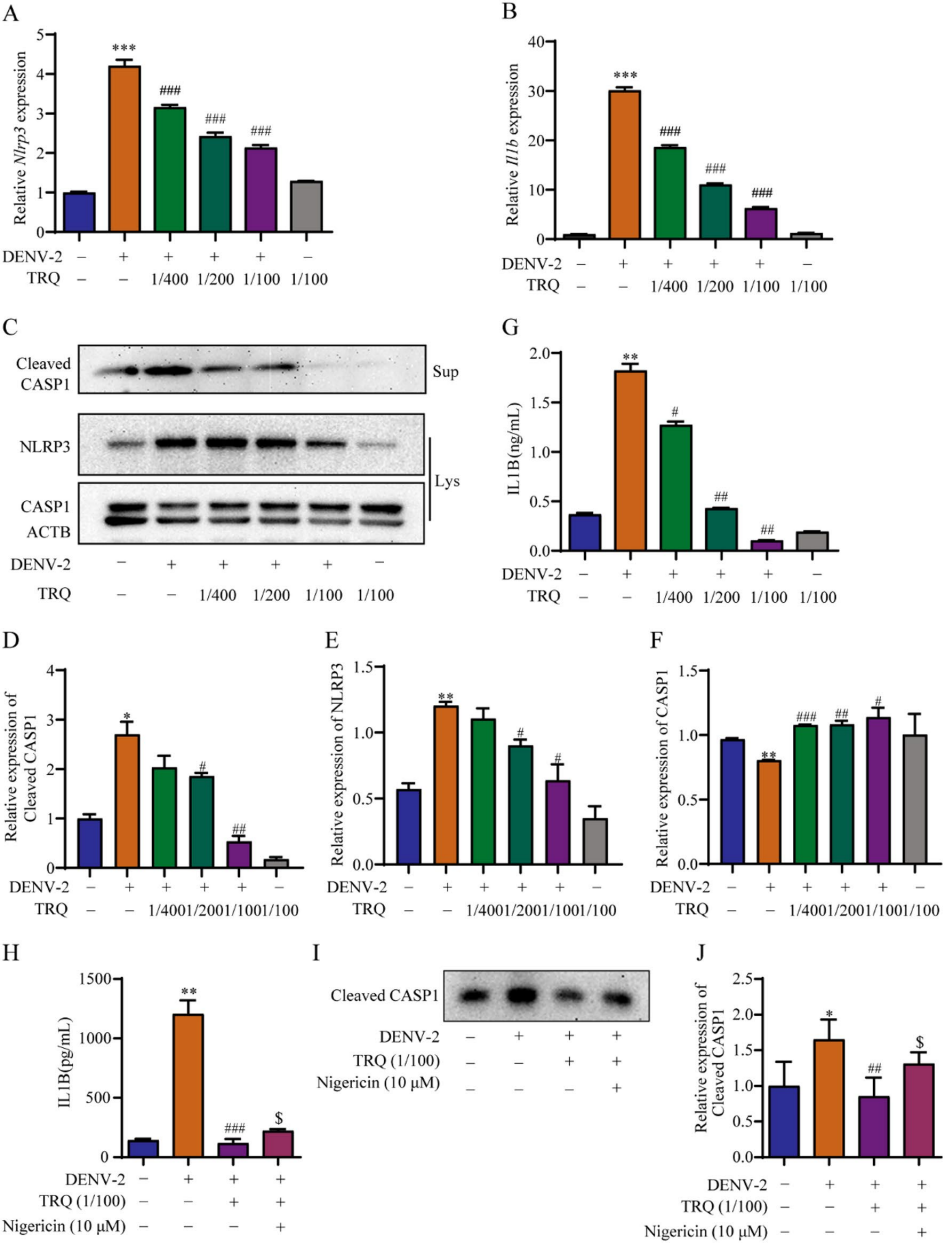
TRQ decreases the activation of NLRP3 in DENV-2 infected BV2 cells. BV2 cells were infected with DENV-2 (0.5 MOI) in the presence or absence of TRQ (1/400, 1/200, 1/100) for 24 h. **A**, **B** TRQ downregulates the mRNA expression levels of *Nlrp3* (**A**) and *Il1b* (**B**) in DENV-2 infected BV2 cells, as detected by qRT‒PCR. **C**–**F** TRQ decreases the protein expression levels of NLRP3 and cleaved caspase-1 in DENV-2 infected BV2 cells. Total proteins and supernatant proteins were extracted, and Western blotting was performed. **G** TRQ attenuates the release of IL1B in DENV-2 infected BV2 cells. The cell culture media were collected, and ELISA was performed to quantify the concentration of IL1B. **H**–**J** The activation of NLRP3 reverses the anti-inflammatory activities of TRQ in DENV-infected BV2 cells. BV2 cells were infected with DENV-2 (0.5 MOI) in the presence or absence of TRQ (1/100) for 22 h and then treated with 10 µM NLRP3 agonist nigericin for 2 h. The levels of IL1B (**H**) and cleaved CASP1 (**I**, **J**) in supernatants were analyzed by ELISA or Western blotting, respectively.^*^P<0.05,  ^**^P < 0.01, ^***^P < 0.001 *vs.* control group; ^#^*P* < 0.05, ^##^*P* < 0.01, ^###^*P* < 0.001 *vs.* DENV-2 group. ^$^*P* < 0.05 *vs.* DENV-2 + TRQ group

NLRP3 is essential for the activation of inflammasomes in macrophages [42]. Arboviral infections, such as chikungunya and Zika, can activate NLRP3 inflammasome myeloid cells, including macrophages, leading to increased production of IL1B and contributing to pathological inflammatory events that drive the development of disease [43]. Therefore, we evaluated whether TRQ inhibits Nlrp3 inflammasome activation in DENV-2-infected BV2 cells by qRT‒PCR analysis. As shown in Fig. 3A–B, the mRNA expression of *Nlrp3* and *Il1b* in DENV-2 infected BV2 cells obviously increased, while TRQ remarkably restrained the expression of *Nlrp3* and *Il1b*. Western blot analysis clearly revealed that DENV infection obviously increased the protein expression levels of NLRP3 and cleaved CASP1, which phenomena was obviously suppressed by TRQ (Fig. 3C-F). Additionally, TRQ dose-dependently inhibited the release of IL1B in DENV-2 infected BV2 cells (Fig. 3G). To further elucidate the role of NLRP3 in the anti-inflammatory effects of TRQ, we utilized nigericin (an NLRP3 activator) to upregulate the expression of NLRP3. As confirmed by ELISA and Western blotting, nigericin at nontoxicity (Additional file 1: Fig. S2) increased the secretion of IL1B (Fig. 3H) and cleaved CASP1 (Fig. 3I, J), suggesting that nigericin reversed the anti-inflammatory activities of TRQ in DENV-2 infected BV2 cells. These results revealed that TRQ decreases the activation of NLRP3, thereby inhibiting the release of IL1B in DENV-2 infected BV2 cells.

### TRQ protects mouse hippocampal neuronal HT22 cells by decreasing IL1B in the supernatants of DENV-2 infected BV2 cells

**Fig. 4:**
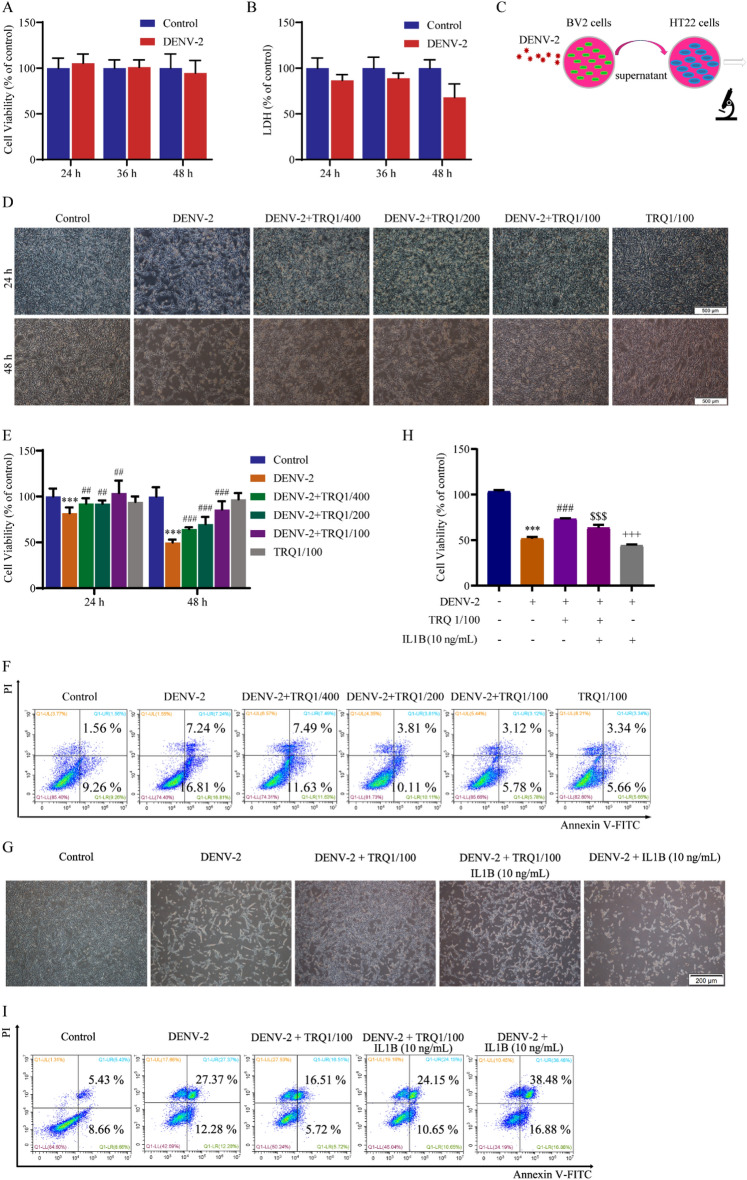
TRQ protects mouse hippocampal neuronal HT22 cells by decreasing IL1B from DENV-2-stimulated BV2 cells. **A**, **B** DENV-2 shows no obvious toxicities in HT22 cells. HT22 cells were incubated with DENV-2 for 24, 36 or 48 h and then examined by CCK-8 (**A**) and LDH (**B**) assays. **C** Steps of adoptive culture. BV2 cells were infected with DENV-2 (0.5 MOI or 5 MOI) in the presence or absence of TRQ for 24 h. Then, HT22 cells were incubated with cell-free supernatants from BV2 cells for 24 or 48 h. **D**, **E** TRQ protects HT22 cells from DENV-2-stimulated BV2 cells supernatants induced death. After adoptive culture, the morphological change and the cell viability of HT22 cells were examined by microscopy (40 ×) (**D**) and CCK-8 assay (**E**). **F** TRQ inhibits apoptosis in HT22 cells induced by supernatants from DENV-2-stimulated BV2 cells. After adoptive culture, the number of apoptotic HT22 cells was analyzed by PI/Annexin V-FITC staining. **G**–**I** IL1B counteracts the protective effect of TRQ. BV2 cells were infected with DENV-2 (0.5 MOI) in the presence or absence of TRQ for 24 h. Then, HT22 cells were incubated with cell-free supernatants from BV2 cells in the presence or absence of IL1B for 48 h. Morphological observation of cell viability and apoptosis of the HT22 cells were performed by microscopy (40 ×) (**G**), CCK-8 assay (**H**) and PI/Annexin V-FITC staining (**I**). ^***^P < 0.001 *vs*. control group, ^##^*P* < 0.01, ^###^*P* < 0.001 *vs*. DENV-2 group, ^$$$^*P* < 0.001 *vs*. DENV-2 + TRQ group, ^+++^*P* < 0.001 *vs*. DENV-2 + TRQ + IL1B group

Due to the important role of neurons in neuroinflammation, we examined the effects of DENV-2 on HT22 neuronal cells by CCK-8 and LDH assays. However, the data showed that DENV-2 had no obvious toxicity in HT22 cells for 24, 36 and 48 h (Fig. 4A–B). We speculated that soluble inflammatory mediators excreted by infected BV2 cells could cause the death of HT22 cells. Thus, HT22 cells were incubated with supernatants from DENV-2 infected BV2 cells [44] (Fig. 4C). As shown in Fig. 4D, TRQ counteracted the decrease of HT22 cells caused by the supernatants of DENV-2 infected BV2 cells at 24 h and 48 h. The same phenomenon was also observed in the cell viability data (Fig. 4E). Apoptosis is a form of programmed cell death. Next, we examined the effect of infected BV2 cell supernatants on the induction of apoptosis in HT22 cells by PI/Annexin V-FITC staining. Our results showed that TRQ decreased the percentage of apoptotic HT22 cells in a dose-dependent manner (Fig. 4F). Because TRQ decreased the activation of NLRP3 and inhibited the release of IL1B in DENV-2 infected BV2 cells, we speculated that the secretion of IL1B is a critical factor that induces the death of HT22 cells. Therefore, we used IL1B to treat HT22 cells in the presence of supernatants of DENV-2 infected BV2 cells after TRQ treatment. Our results showed that co-treatment with IL1B and DENV-2 further decreased the cell viability compared with DENV-2 alone group, while TRQ treatment significantly elevated the cell survival and inhibited the apoptosis of HT22 cells (Fig. 4G–I). These results reveal that TRQ protects HT22 cells from DENV-2 induced death by decreasing the secretion of IL1B in DENV-2 infected BV2 cells.

### TRQ attenuated encephalitis in DENV-2 infected ICR mice

**Fig. 5:**
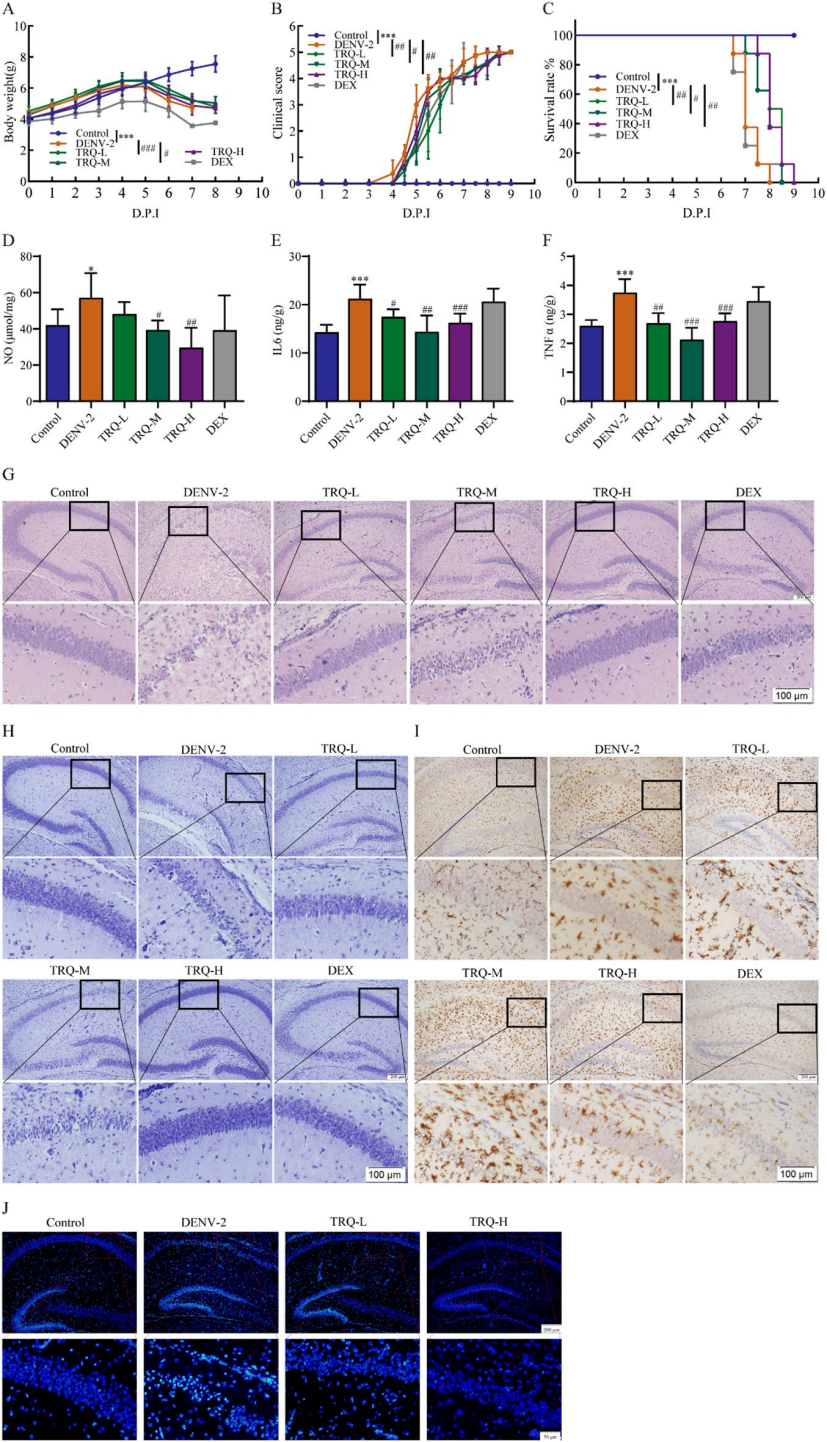
TRQ inhibits encephalitis in DENV-infected ICR suckling mice. Seven-day-old ICR suckling mice were infected with DENV-2 by intracranial injection, and TRQ (1.25, 2.5, 5.0 mL/kg) was administered by intraperitoneal injection. The body weights (**A**), disease manifestations (**B**) and survival rates (**C**) of mice were monitored daily (*n* = 6). Disease manifestations were scored as follows: 0 for health, 1 for minor symptoms (reduced mobility and hunched posture), 2 for limbic seizure, 3 for moving difficulties (anterior or posterior limb weakness), 4 for paralysis and 5 for death. **D**–**F** TRQ inhibits the release of NO, IL6 and TNFα. After mice were sacrificed, the levels of NO, IL6 and TNFα in the brain were measured by NO or ELISA kits (*n* = 6). **G** TRQ ameliorates the brain tissue lesions caused by DENV-2. The brain tissues were dehydrated and embedded in paraffin. After sectioning a thickness of 4 μm, brain pathological changes were detected by HE staining (100 ×). **H** TRQ decreases neuronal injury caused by DENV-2 detected by Nissl staining (100 ×). **I** TRQ inhibits the microglial activation in DENV-infected ICR suckling mice, as examined by immunohistochemistry. Representative image of Iba-1 immunostaining of Iba-1 in the brain (100 ×). **J** TRQ reduces DENV-2-induced apoptosis in neurons. Apoptosis was detected by Hoechst 33258 staining (100 ×). ^*^P < 0.05, ^***^P < 0.001 *vs*. control group, ^#^*P* < 0.05, ^##^*P* < 0.01, ^###^*P* < 0.001 *vs*. DENV group

To evaluate the therapeutic potential of TRQ against DENV-2 infection in vivo, an encephalitis model was established in ICR suckling mice as previously reported [39, 45]. As shown in Fig. 5A–C, TRQ alleviated the trend toward weight loss, decreased the clinical scores and prolonged the survival time of DENV-2 infected ICR mice.

Next, the inflammatory mediators in the brains of in infected ICR suckling mice were detetced by ELISA analysis. Our results demonstrated that compared with the model group, TRQ decreased the secretion of NO, IL6 and TNFα compared to the model group (Fig. 5D–F). DENV-2 infected mice exhibited neuronal loss in the hippocampus, which was alleviated by TRQ (Fig. 5G). Further Nissl staining confirmed neuronal cell loss in DENV-infected mice, while TRQ reduced the death of neurons (Fig. 5H). Microglia can be targeted for activation by DENV infection in vivo [39, 46]. The activation of microglia in the brain is an important mechanism during neuroinflammatory processes, including encephalitis. Iba-1 (a marker of microglial activation) was utilized to evaluate microglial activation. In DENV-2 infected mouse brain tissues, many brown cells were observed, indicating the activation of microglia. However, TRQ reduced these changes (Fig. 5I). Moreover, TRQ reduced neuronal apoptosis in DENV-2 infected ICR suckling mice as reflected by Hoechst 33258 staining data (Fig. 5J). These findings suggest the protective effects of TRQ on DENV-2 infected mice.

### TRQ inhibits the activation of NLRP3 in the brains of DENV-2 infected ICR mice

**Fig. 6:**
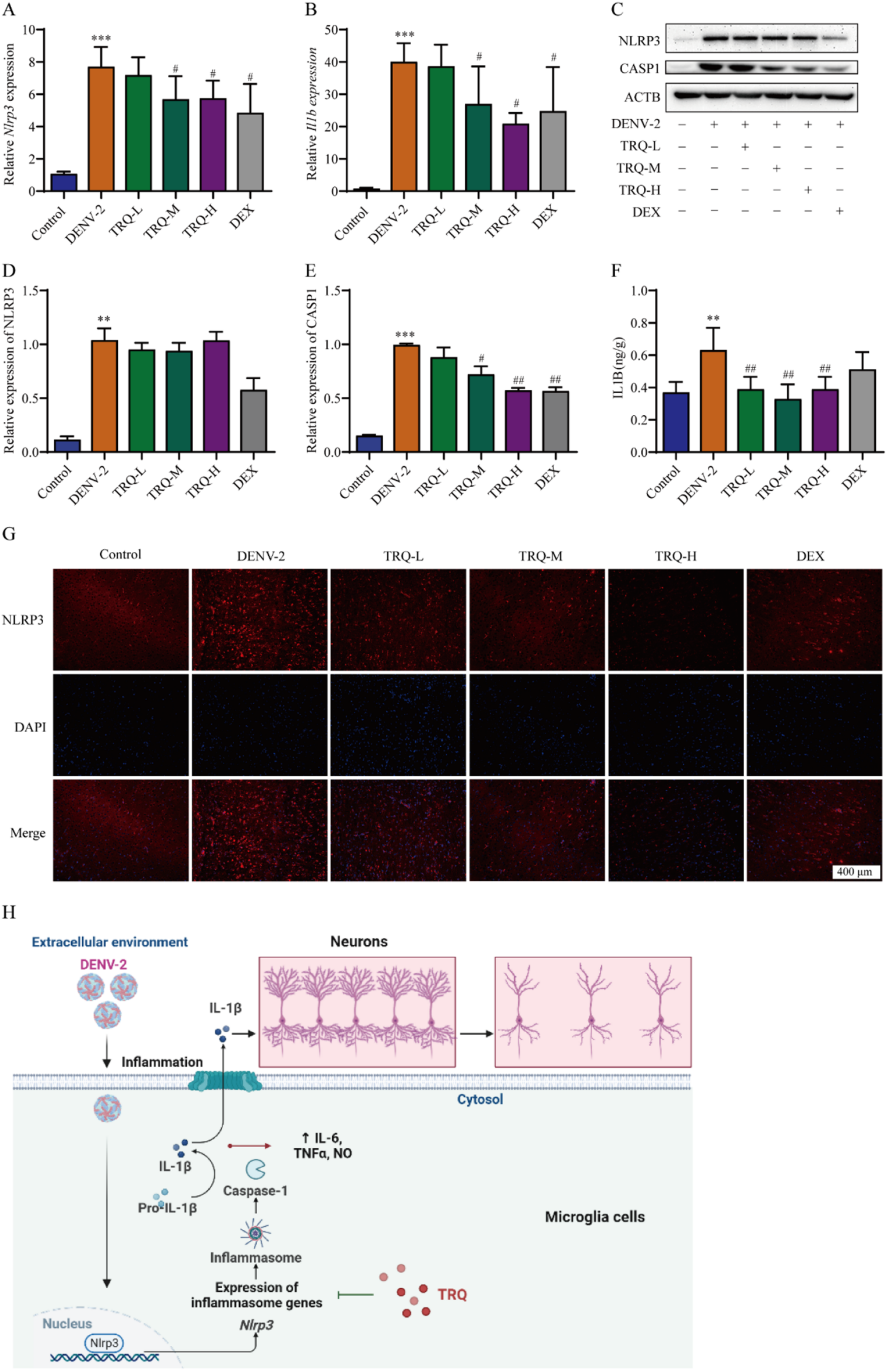
TRQ inhibits the activation of NLRP3 in brain tissues of DENV-infected mice. **A**, **B** TRQ decreases the mRNA expression levels of *Nlrp3* (**A**) and *Il1b* (**B**) in DENV-infected mice (*n* = 6). The brain tissues of the mice were harvested and total mRNAs were extracted and analyzed by qRT‒PCR. **C**–**E** TRQ decreases the protein expression level of CASP1 in DENV-infected mice. The brain tissues of the mice were harvested and total protein lysates were collected and analyzed by Western blotting. **F** TRQ decreases the release of IL1B in DENV-infected mice. The level of IL1B in the brain was measured by ELISA (*n* = 6). **G** TRQ inhibits NLRP3 in DENV-infected mice. The brain tissues were sectioned and the expression of NLRP3 in the mouse brain tissues was determined with anti-NLRP3 antibodies by immunofluorescence staining (200 ×). DAPI was used to stain the nucleus. ^**^P < 0.01, ^***^P < 0.001 *vs*. control group, ^#^*P* < 0.05, ^##^*P* < 0.01 *vs*. DENV group. (**F**) The mechanism of TRQ on dengue encephalitis. The schematic was drawn with BioRender (https://biorender.com/)

To further confirm the role of NLRP3 in TRQ against DENV encephalitis, we detected the NLRP3 and IL1B expression levels in DENV-2 infected ICR mice. As shown in Fig. 6A–B, DENV-induced *Nlrp3* and *Il1b* mRNA expression was inhibited by TRQ. The inhibitory effect of TRQ was also confirmed by Western blot analysis (Fig. 6C–E). The protein levels of CASP1 showed a dose-dependent decrease by TRQ. Moreover, the elevated expression of IL1B in DENV-2 infection group was significantly decreased upon TRQ treatment (Fig. 6F). Finally, immunofluorescence analysis revealed that TRQ inhibited the activation of NLRP3 in brain tissues of DENV-2 infected ICR mice (Fig. 6G). Collectively, these data suggest that TRQ displayed potential therapeutic effect in DENV encephalitis by alleviating microglia-mediated inflammation in vitro and in vivo.

## Discussion

Recent anti-DENV candidates with potential clinical application mainly include compounds inhibiting DENV entry and targeting viral NS proteins and host factors [47]. However, no definitive and effective antiviral drugs are available for dengue infection treatment. TRQ was approved for use by the Chinese Food and Drug Administration in 2003. Because of its anti-inflammatory, antiviral, and antibacterial effects, TRQ has been used to treat acute cerebral ischemia, acute cholecystitis, and infantile acute pneumonia for many years. According to the Guidelines for the Diagnosis and Treatment of Dengue Fever (second edition, 2014), TRQ has been included in the treatment scheme of traditional Chinese medicine (TCM) based on syndrome differentiation. In the Guidelines for the Diagnosis and Treatment of Dengue Fever, dengue encephalitis is classified as severe dengue fever. Hence, this study was conducted to determine the inhibitory effects of TRQ on DENV encephalitis. We firstly present the quantification of 7 compounds in TRQ, including chlorogenic acid, protocatechuic acid, caffeic acid, baicalin, ursodeoxycholic acid, chenodeoxycholic acid and forsythin. These compounds all display anti-inflammatory effects to treat various inflammation-related diseases [48–54].

DENV is transmitted principally by *Aedes aegypti* and *Aedes albopictus*. In addition, baby hamster kidney cells BHK-21 are extensively used to evaluate the antiviral activities of drugs. Therefore, C6/36 cells and BHK-21 cells were selected for virus propagation and antiviral efficacy evaluation, respectively. Rodent species are more susceptible to DENV NGC strain infection than human cells, as demonstrated by obvious cytopathic effect and high viral copy numbers. We next evaluated the protective effects of TRQ on BHK-21 cells after DENV infection by using MTT assay. However, our results showed no significant difference in absorbance values between the DENV infection and control groups, which may be due to the reaction between the DENV-2 strain used in this study and MTT solution. Therefore, we next used the CCK8 assay to measure the cell viability after DENV infection. Our results revealed that co-treatment with DENV and TRQ significantly elevated the survival rate of BHK-21 cells, while treatment post DENV infection had no significant effects. These results demonstrate that TRQ exerts anti-DENV effects at viral binding and entry stage rather than at post-entry stage. Plaque formation assays showed that TRQ inhibited DENV-2 binding to cells and alleviated the plaques formed by DENV-2 progeny virus. In addition, TRQ significantly reduced the viral load in brain tissue of DENV-2 infected mice at high dosages. However, all doses of TRQ show favourable therapeutic effects in vivo. This confirms the multi-component, multi-target and multi-pathway action characteristics of TCM. Considering the poor anti-DENV-2 activity and significant anti-inflammatory activity of TRQ, we explored whether inflammation is involved in the alleviating effect of TRQ on dengue encephalitis.

Viral encephalitis is an infectious disease of the CNS caused by a variety of viral infections. Viruses can directly damage glial and neuronal cells, while the immune inflammatory response during viral infection also plays a key role in neuronal damage. Inflammatory changes in the meninges and brain parenchyma are the main pathological features, including glial cell activations and neuronal necrosis. In addition to the destruction of nerve cells by the virus itself, immune-mediated inflammation in the acute phase of viral infection is an important way to cause brain tissue damage. Virus-induced secretion of cytokines, such as TNF and IL, contributes to innate immunity against viral infection. The pathogenesis of dengue fever is unclear, but the large amount of cytokine secretion (cytokine storm) is thought to be one of the main pathogenic factors [55, 56]. According to our study, TRQ attenuated the expression of proinflammatory cytokines IL-6, TNF-α and NO both in vitro and in vivo relative to the DENV group, indicating that TRQ can remarkably restrain DENV-induced inflammation. Our study comprehensively evaluated the role of TRQ as an anti-inflammatory agent against DENV for the first time.

Inflammasomes, including NLRP3, are a group of multiprotein cytoplasmic receptors that sense pathogen-related and hazard-related molecular patterns in response to pathogen infection and cell injury. Then, the activation of caspase-1 is triggered and consequently leads to the maturation of inflammatory cytokines such as IL1B. The NLRP3 inflammasome is associated with a variety of human diseases, including Alzheimer’s disease, obesity, rheumatoid arthritis, asthma, nonalcoholic fatty liver disease, and autoimmune encephalitis [57]. DENV induced IL1B activation in blood samples from infected patients and macrophages in mice [11]. Although NLRP3 can be activated in the periphery of DENV-infected patients, the role of the NLRP3 inflammasome in DENV encephalitis has not been reported. Therefore, studying the relationship between the NLRP3 inflammasome and DENV encephalitis is of great significance for the biological basis of disease occurrence and development as well as drug development. We demonstrated that TRQ effectively inhibited DENV-induced injuries by suppressing NLRP3 and decreasing the release of IL1B in vitro and in vivo. Reports have demonstrated that IL1B disrupts the blood–brain barrier and peripheral immune cells infiltrate the CNS and aggravate inflammation of the nervous system. At the same time, IL1B activates microglia, which in turn induces IL6, TNFα and other inflammatory factors and further induces the synthesis of nitric oxide synthase and NO production [58, 59]. It has been reported that DENV M protein triggers NLRP3 inflammasome activation and IL1B secretion due to the interaction of M protein with NLRP3 [11]. However, whether TRQ could suppress the process warrants more investigation. TRQ also showed a dose-dependent inhibition effect on the release of CASP1 in vivo. CASP1 is a crucial indicator for detecting cell pyroptosis [60]. The NLRP3 inflammasome activate by DENV can modify the CASP1 and the growth of cytokines. Therefore, tissue damage will be relieved in disease if these signals are blocked [61]. TRQ may reduce brain damage by inhibiting CASP1 and NLRP3 inflammasome.

The immune inflammatory response of host cells caused by virus infection plays a key role in neuronal injury. The production of proinflammatory cytokines after microglial cell activation in DENV infection could trigger neuronal cell death [62]. In this study, HT22 cells were not damaged by DENV-2 directly but injured by the supernatant of DENV-2 infected BV2 cells. However, this damage was decreased after TRQ treatment. Moreover, additional IL1B supplementation reversed the protective effect of TRQ. These results suggested that other factors, such as inflammatory molecules induced by DENV, alter the states of glial cells and stimulate the death of neurons.

DENV infection can result in neuronal loss and glial activation. Our results showed that both microglial activation and neuronal damage caused by DENV-2 were mitigated by TRQ in ICR suckling mice. Treatment with TRQ increased the average body weight of ICR suckling mice. The clinical score raised by DENV-2 was alleviated by TRQ. Patients with dengue encephalitis are at risk of death. Our results showed that TRQ delayed the death of DENV-2 infected ICR suckling mice. TRQ significantly reduced brain injury and alleviated the symptoms caused by DENV-2 in vivo. The data from in vivo experiments are of significance for supporting the clinical use of TRQ.

Altogether, this study provides a primary explanation for the hypothesis that TRQ could alleviate DENV-induced encephalitis. TRQ protects against brain tissue damage caused by DENV infection by reducing microglial activation, decreasing inflammatory responses, and protecting neuronal cells (Fig. 6H). Our findings are not only helpful for related drug discovery and clinical use of TRQ but also further prove that TCM used to treat infection-related diseases may be closely associated with its anti-inflammatory activities.

### Supplementary Information

**Additional file 1: Fig. S1.** DENV-2 activates the mRNA expression levels of *iNOS, Il6* and *Tnfα* in BV2 cells. After exposed to DENV-2 (0.5 MOI), the mRNA level of *iNOS* (**A**), *Il6* (**B**) and *Tnfα* (**C**) were analyzed by qRT‒PCR. ^***^P < 0.001 *vs*. control group. **Fig. S2.** The cytotoxicity of nigericin in BV2 cells. BV2 cells were incubated with nigericin (0.06 μM–10 μM) for 12 h. The cell viability was determined by MTT assay. **Fig. S3.** **A** TRQ downregulates the protein expression level of E protein in DENV-2 infected mouse brains, as examined by Western blotting. **B** TRQ decreases the protein expression levels of NLRP3 and cleaved CASP1 in DENV-2 infected BV2 cells. Total proteins and supernatant proteins were extracted, and Western blotting was performed. **C** The activation of NLRP3 reverses the anti-inflammatory activities of TRQ in DENV-infected BV2 cells. BV2 cells were infected with DENV-2 (0.5 MOI) in the presence or absence of TRQ (1/100) for 22 h and then treated with 10 µM NLRP3 agonist nigericin for 2 h. The levels of cleaved CASP1 in supernatants were analyzed by Western blotting. **D** TRQ decreases the protein expression level of CASP1 in DENV-infected mice. The brain tissues of the mice were harvested and total protein lysates were collected and analyzed by Western blotting.

## Data Availability

The data produced from this study are available from the first author and the corresponding author upon reasonable request.

## Abbreviations

BSL-3 laboratory: Biosafety level 3 laboratory
CASP1/Caspase-1: Cysteinyl aspartate specific proteinase-1
CCK-8: Cell counting kit-8
DAPI: 4ʹ, 6-Diamidino-2-phenylindole
DENV: Dengue virus
DMSO: Dimethyl sulfoxide
ECL: Enhanced chemiluminescence
ELISA: Enzyme-linked immunosorbent assay
H&E: Hematoxylin and eosin
IL1B/IL-1β: Interleukin 1 beta
IL6: Interleukin 6
MTT: 3-[4, 5-Dimethylthiazol-2-yl]-2,5-diphenyltetrazoliumbromide
NLRP3: NOD-like receptor thermal protein domain associated protein 3
PBS: Phosphate-buffered saline
TNFα: Tumor necrosis factor-alpha
TRQ: Tanreqing injection
UPLC-MS/MS: Ultra-high performance liquid chromatography combined with triple quadrupole mass spectrometry
