# Supplementary material for: Tanreqing injection inhibits dengue virus encephalitis by suppressing the activation of NLRP3 inflammasome
Source: Chin Med. 2024 Feb 14;19:24. doi: 10.1186/s13020-024-00893-2 (PMC10868054; doi:10.1186/s13020-024-00893-2)
Supplement: Supplementary file 1 — Additional file 1: Fig. S1. DENV-2 activates the mRNA expression levels of iNOS, Il6 and Tnfα in BV2 cells. After exposed to DENV-2 (0.5 MOI), the mRNA level of iNOS (A), Il6 (B) and Tnfα (C) were analyzed by qRT‒PCR. ***P < 0.001 vs. control group. Fig. S2. The cytotoxicity of nigericin in BV2 cells. BV2 cells were incubated with nigericin (0.06 μM–10 μM) for 12 h. The cell viability was determined by MTT assay. Fig. S3. A TRQ downregulates the protein expression level of E protein in DENV-2 infected mouse brains, as examined by Western blotting. B TRQ decreases the protein expression levels of NLRP3 and cleaved CASP1 in DENV-2 infected BV2 cells. Total proteins and supernatant proteins were extracted, and Western blotting was performed. C The activation of NLRP3 reverses the anti-inflammatory activities of TRQ in DENV-infected BV2 cells. BV2 cells were infected with DENV-2 (0.5 MOI) in the presence or absence of TRQ (1/100) for 22 h and then treated with 10 µM NLRP3 agonist nigericin for 2 h. The levels of cleaved CASP1 in supernatants were analyzed by Western blotting. D TRQ decreases the protein expression level of CASP1 in DENV-infected mice. The brain tissues of the mice were harvested and total protein lysates were collected and analyzed by Western blotting. [file 13020_2024_893_MOESM1_ESM.docx]

**Supporting Information**

**Fig. S1**


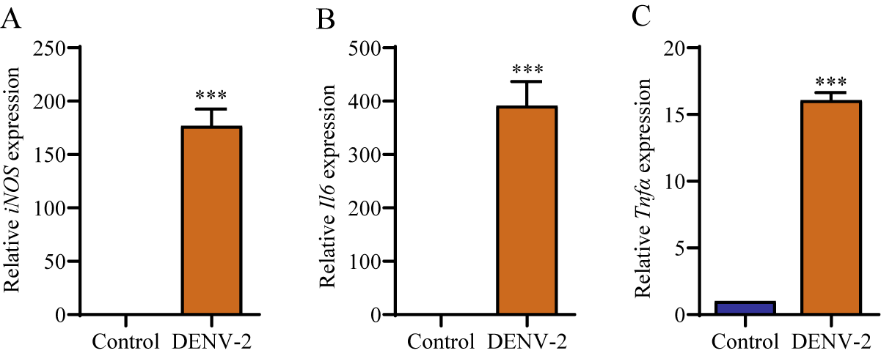


**Fig. S1.** DENV-2 activates the mRNA expression levels of *iNOS, Il6* and *Tnfα* in BV2 cells. After exposed to DENV-2 (0.5 MOI), the mRNA level of *iNOS* (A), *Il6* (B) and *Tnfα* (C) were analyzed by qRT‒PCR. ^***^*P* < 0.001 *vs*. control group.

**Fig. S2**


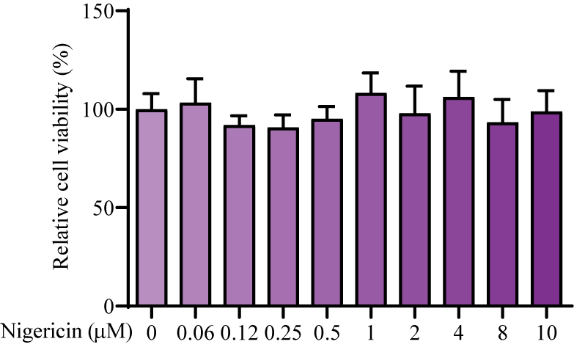


**Fig. S2.** The cytotoxicity of nigericin in BV2 cells. BV2 cells were incubated with nigericin (0.06 μM ~ 10 μM) for 12 h. The cell viability was determined by MTT assay.

**Fig. S3**

**
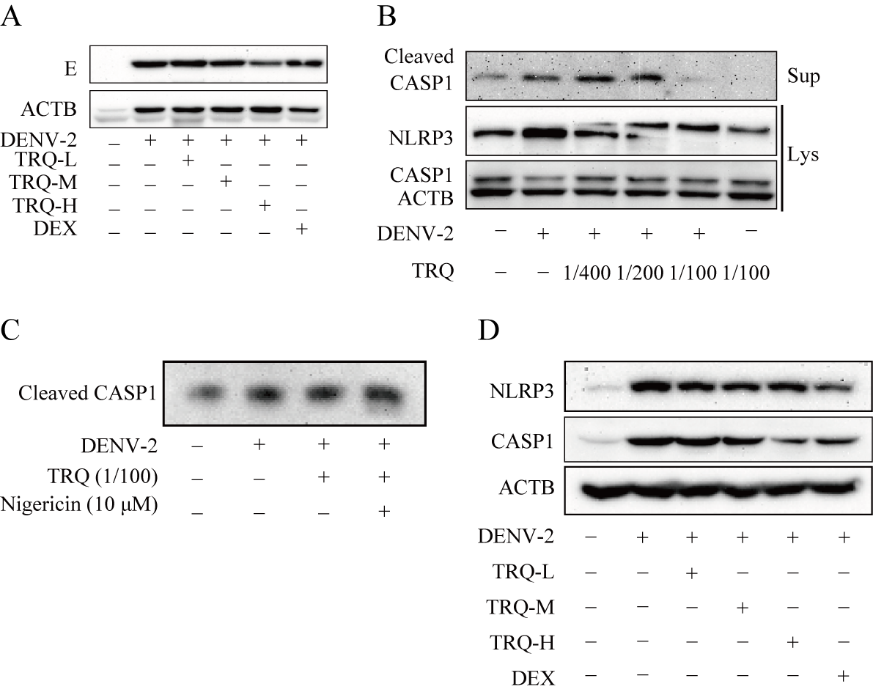
**

**Fig. S3.** (A) TRQ downregulates the protein expression level of E protein in DENV-2 infected mouse brains, as examined by Western blotting. (B) TRQ decreases the protein expression levels of NLRP3 and cleaved CASP1 in DENV-2 infected BV2 cells. Total proteins and supernatant proteins were extracted, and Western blotting was performed. (C) The activation of NLRP3 reverses the anti-inflammatory activities of TRQ in DENV-infected BV2 cells. BV2 cells were infected with DENV-2 (0.5 MOI) in the presence or absence of TRQ (1/100) for 22 h and then treated with 10 µM NLRP3 agonist nigericin for 2 h. The levels of cleaved CASP1 in supernatants were analyzed by Western blotting. (D) TRQ decreases the protein expression level of CASP1 in DENV-infected mice. The brain tissues of the mice were harvested and total protein lysates were collected and analyzed by Western blotting.
